# Assignment of the Crystal Structure to the *Aza*-Pinacol Coupling Product by X-ray Diffraction and Density Functional Theory Modeling

**DOI:** 10.1021/acsomega.2c05446

**Published:** 2022-11-03

**Authors:** Oleksandr Savateev, Nadezda V. Tarakina, Alexander P. Tyutyunnik, Salvador Martinez Rivadeneira, Julian Heske, Thomas D. Kühne

**Affiliations:** † Department of Colloid Chemistry, Max-Planck Institute of Colloids and Interfaces, Research Campus Golm, 14476 Potsdam, Germany; ‡ Institute of Solid State Chemistry, Ural Branch of the Russian Academy of Sciences, 91 Pervomayskaya Street, 620990 Ekaterinburg, Russia; § Dynamics of Condensed Matter and Center for Sustainable Systems Design, Chair of Theoretical Chemistry, University of Paderborn, Warburger Street 100, D-33098 Paderborn, Germany

## Abstract

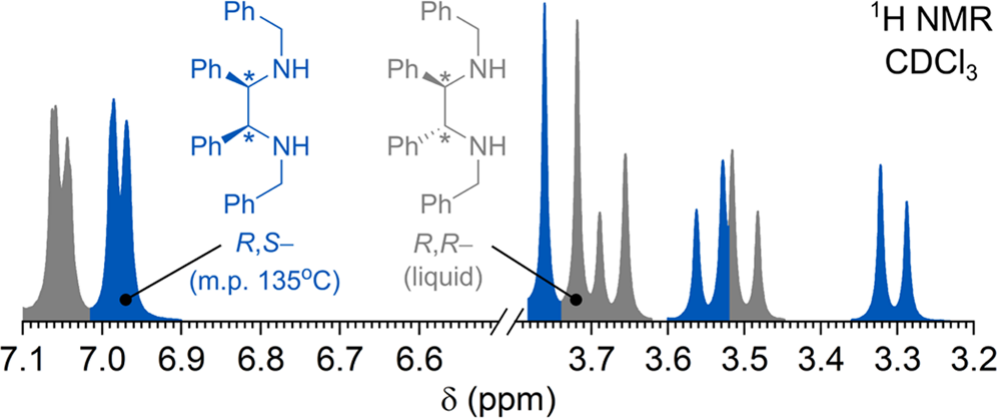

*Aza*-pinacol coupling of *N*-benzyl-1-phenylmethanimine using Zn dust affords a mixture of *R*,*S-* or *R*,*R*-diastereomers in a 1:1 ratio. The *R*,*S*-diastereomer is solid with an m.p. of 135 °C, while the *R*,*R-*diastereomer is liquid at room temperature. The configuration of stereocenters was determined by combining X-ray powder diffraction and density functional theory (DFT) modeling.

## Introduction

**Figure 1:**
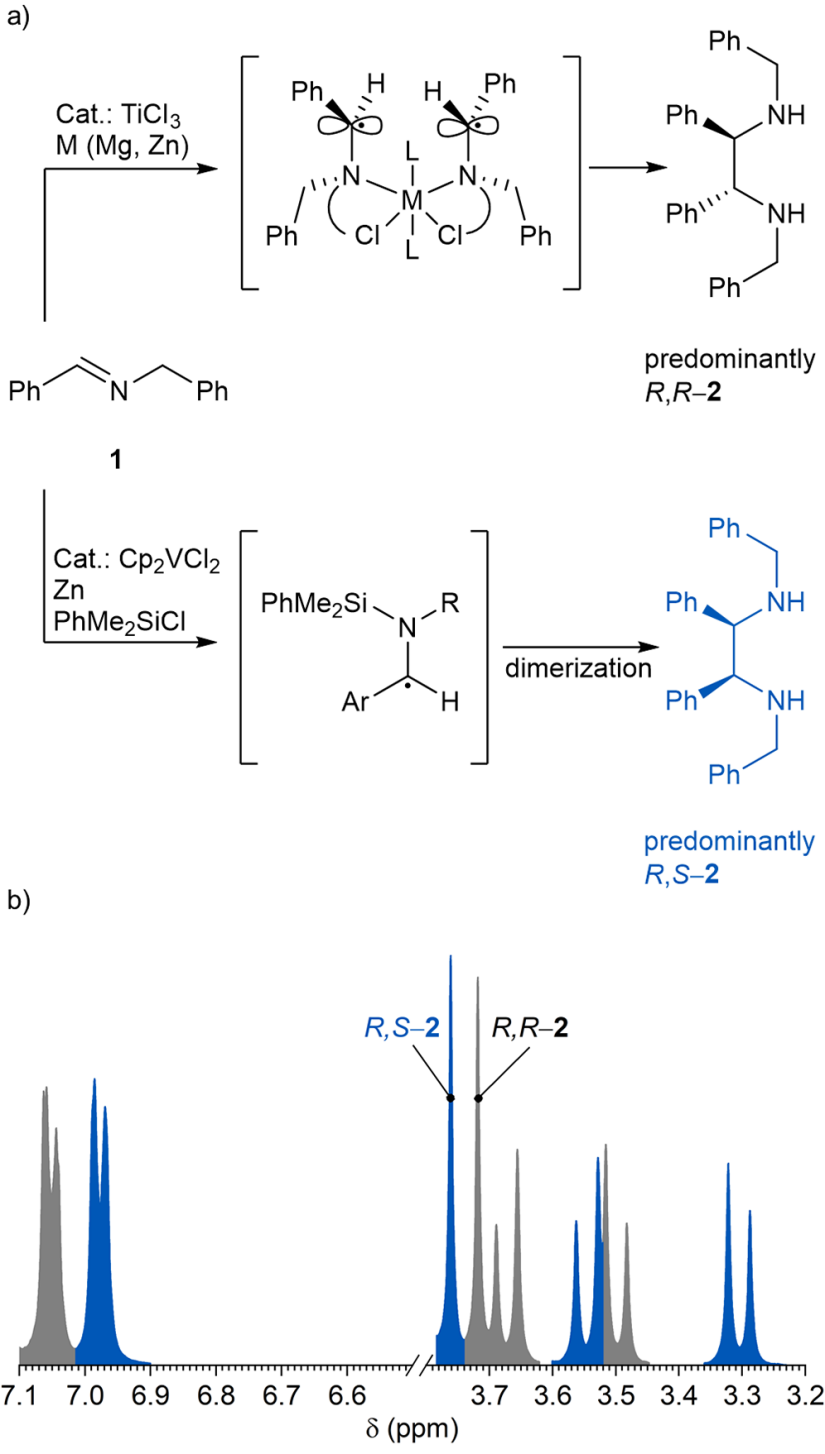
(a) Diastereoselectivity control in *aza*-pinacol coupling of imines. (b) A fragment of the ^1^H NMR spectrum of the *R*,*R***-2** and *R*,*S***-2** (1:1) mixture in CDCl_3_. Peaks were assigned to the corresponding isomers taking into account the findings of this work.

*Aza*-pinacol coupling of imines **1** offers a convenient approach for the synthesis of tetrasubstituted ethanediamines **2** (Figure 1a), which are used, for example, as chiral ligands in catalysis.^1,2^ Zn dust,^3,4^ mischmetall,^5^ Na,^6^ and TiC_4_/amalgamated Mg^7^ have been investigated as reductants. On the other hand, *aza*-pinacol coupling of imines is enabled by photoredox catalysis and sensitizers, such as Ir–polypyridine complex,^8^ heterogeneous CdS semiconductor,^9^ or transition metal-free organic dyes, *N*-phenylphenothiazine,^10^ diphenyldibenzocarbazole,^11^ and perylene.^12^ Typically, *aza*-pinacol coupling gives a mixture of *R*,*S*- and *R*,*R-*isomers, which are often tagged as “*meso*-isomer” (has a plane of symmetry) and “d,l-isomer,” respectively.^7^ However, a combination of a low-valent Ti complex and reductant (Mg, Zn) allows shifting the diastereoselectivity toward predominantly *R*,*R*-isomers due to minimized steric influence in the intermediary complex (Figure 1a). A combination of the Cp_2_VCl_2_ catalyst, Zn as a reductant, and PhMe_2_SiCl as an additive, on the other hand, produces predominantly *R*,*S*-isomers due to the repulsion of the lone pair on nitrogen and steric hindrance between the aryl groups.^3^

The diastereomeric ratio (d.r.) is conveniently determined from the ^1^H NMR spectrum—benzylic CH_2_ protons appear as the AB system, while chemical shifts in the diastereomers differ by ∼0.1 ppm (Figure 1b). Although ^1^H NMR spectroscopy can distinguish between the isomers, it cannot assign the configuration of the stereocenters in **2**. Furthermore, by analyzing ^1^H NMR data, we noticed an inconsistency in the assignment of NMR peaks of the AB system to either *R*,*S*-**2** or *R*,*R***-2** in more recent publications^8,12^ and earlier ones.^7,13,14^

To resolve this discrepancy and provide a reliable reference, we synthesized *R*,*S*-**2** and *R*,*R*-**2** via *aza*-pinacol coupling of imine **1** with Zn dust. In agreement with earlier publications,^7,14^ the diastereomers possess substantially different physical properties—one diastereomer is solid, while another is liquid at room temperature. Due to this feature, the solid diastereomer was conveniently separated by crystallization from MeCN. A combination of X-ray powder diffraction and density functional theory (DFT) modeling allowed us to unambiguously assign *R*,*S-*configuration to the solid, while the *R*,*R*-isomer is liquid at room temperature.

## Experimental Section

### X-ray Powder Diffraction (XRPD)

XRPD patterns for Rietveld refinement were collected at room temperature on a transmission STADI-P (STOE, Germany) diffractometer equipped with a linear mini-PSD detector using Cu Kα_1_ radiation in the 2θ range of 5–120° with a step of 0.02°. Polycrystalline silicon (*a* = 5.43075(5) Å) was used as an external standard. The crystal structure was solved based on the XRPD data using the EXPO2014 program^15^ and refined employing GSAS software.^16,17^ The peak profiles were fitted with a pseudo-Voigt function *I*(2θ) = x*L(2θ) + (1 – *x*)*G(2θ) (where *L* and *G* are the Lorentzian and Gaussian parts, respectively). The angular dependence of the peak width was described by the relation (FWHM)^2^ = *U*tg^2^θ + *V*tgθ + *W*, where FWHM is the full line width at half-maximum. The background level was described by a combination of 36th-order Chebyshev polynomials. The absorption correction function for a flat-plate sample in transmission geometry was applied.

### Transmission Electron Microscopy (TEM)

For TEM observations, the sample was crushed in an agate mortar without using any solvent, and then distributed on a Cu grid with a holey carbon support. The TEM study was performed using a double Cs-corrected JEOL JEM-ARM200F (S)TEM operated at 80 kV and equipped with a cold-field emission gun and a Gatan Quantum GIF spectroscopy system.

### Differential Scanning Calorimetry (DSC)

Measurements were performed using Netzsch DSC204 equipped with TASC 414/4 and CC200L controllers under nitrogen flow at a heating rate of 5 K min^–1^.

### Fourier Transform Infrared Spectroscopy (FT-IR)

FT-IR spectra were acquired in the attenuation total reflection mode using a Thermo Scientific Nicolet iD5 spectrometer.

### NMR Spectroscopy

^1^H and ^13^C NMR spectra were recorded on an Agilent 400 MHz (at 400 MHz for Protons and 101 MHz for Carbon-13). Chemical shifts are reported in ppm versus the solvent residual peak as an internal standard. In ^1^H NMR spectra, the peak at 7.26 ppm belongs to CHCl_3_ and that at 1.94 ppm belongs to CH_3_CN. In ^13^C NMR spectra, the peak at 77.2 ppm belongs to CDCl_3_ and that at 1.3 ppm belongs to CD_3_CN.

### High-Resolution Mass Spectroscopy

High-resolution mass spectral data were obtained using a Waters XEVO G2-XS QTOF with an Acquity H-Class (HPLC).

### Density Functional Theory Calculations

Periodic density functional theory calculations were conducted employing the hybrid Gaussian and plane wave approach, as implemented in the CP2K/Quickstep code.^18^ Therein, the charge density was represented by plane waves with a density cutoff of 500 Ry, whereas the Kohn–Sham orbitals were described by an accurate molecularly optimized double-zeta basis set with one additional set of polarization functions.^19^ The B97-D exchange and correlation functional plus a damped pairwise dispersion correction to account for long-range London dispersion forces was used.^20^ Separable dual-space norm-conserving pseudopotentials were employed to mimic the interactions between the valence electrons and the nuclear cores.^21^ The parameters of the supercell, which contains two molecules of the respective monomer, are *a* = 14.82 Å, *b* = 5.38 Å, *c* = 14.04 Å, α = γ = 90.0°, and β = 95.7°. Optimized geometries were obtained by minimizing with respect to its atomic positions by dynamical simulated annealing based on the second-generation Car–Parrinello method of Kühne et al.^22,23^ The coordinates of the eventual optimized structure are provided in Table S1 in the Supporting Information.

#### Synthesis of Imine **1**

A solution of benzaldehyde (10.6 g, 0.1 mmol) and benzylamine (10.7 g, 0.1 mmol) in EtOH (45 mL) was stirred at reflux for 1.5 h. The solvent was concentrated in vacuum (+50 °C, 5 mbar).

Yield: 99%, pale yellow oil. ^1^H NMR (400 MHz, CD_3_CN) δ 8.46 (t, *J* = 1.5 Hz, 1H), 7.80–7.75 (m, 2H), 7.48–7.41 (m, 3H), 7.35 (d, *J* = 4.4 Hz, 4H), 7.30–7.25 (m, 1H), 4.77 (d, *J* = 1.6 Hz, 2H). ^13^C NMR (101 MHz, CD_3_CN) δ 162.7, 140.8, 137.4, 131.7, 129.6, 129.4, 129.0, 128.9, 127.8, 65.5.

#### Synthesis of *R*,*S*-**2** and *R,R*-**2**

*R*,*S*-**2** and *R*,*R*-**2** were prepared according to the adapted procedure.^4^ Zn dust (24.9 g, 383 mmol) was added in portions to a stirred mixture of imine **1** (4.88 g, 25 mmol) in an aqueous solution of NaOH (1.3 M, 100 mL). The reaction mixture was stirred at room temperature overnight. The solid was separated by filtration followed by washing with EtOAc. The organic phase was separated, dried over anhydrous Na_2_SO_4_, and concentrated in vacuum. The residue was triturated with MeCN, the solid was filtered, washed with a small amount of MeCN, and dried on a filter to give *R*,*S*-**2** as a white solid with an m.p. of 135 °C. Yield: 1.04 g, 21%. The solution was concentrated in vacuum (+50 °C, 100 mbar). Dibenzylamine was distilled in vacuum (0.03 mbar, 100 °C). The residue was purified by flash column chromatography on basic Al_2_O_3_ (130 g) by increasing the polarity of the mobile phase from hexane/EtOAc (19:1, 36 × 30 mL) to hexane/EtOAc (9:1, 8 × 30 mL). Yield of *R*,*R*-**2**: 0.72 g (oil), 15%.

*R*,*S*-**2**: ^1^H NMR (400 MHz, CDCl_3_) δ 7.35–7.25 (m, 10H), 7.23–7.15 (m, 6H), 6.98–6.92 (m, 4H), 3.75 (s, 2H), 3.53 (d, *J* = 13.7 Hz, 2H), 3.29 (d, *J* = 13.8 Hz, 2H), 1.82 (s, 2H). ^13^C NMR (101 MHz, CDCl_3_) δ 140.7, 140.3, 128.7, 128.5, 128.3, 128.0, 127.8, 126.8, 67.2, 51.0. High-resolution mass spectrometry (HRMS) (*m*/*z*): [M + H]^+^ calcd for C_28_H_29_N_2_^+^, 393.2325; found, 393.2304.

*R*,*R*-**2**: ^1^H NMR (400 MHz, CDCl_3_) δ 7.33–7.22 (m, 10H), 7.19–7.14 (m, 6H), 7.07–7.05 (m, 4H), 3.76 (s, 2H), 3.71 (d, *J* = 13.1 Hz, 2H), 3.53 (d, *J* = 13.3 Hz, 2H), 2.51 (br. s., 2H). ^13^C NMR (101 MHz, CDCl_3_) δ 140.3, 140.1, 128.5, 128.3, 128.2, 128.1, 127.3, 127.1, 68.2, 51.3. HRMS (*m*/*z*): [M + H]^+^ calcd for C_28_H_29_N_2_^+^, 393.2325; found, 393.2309.

## Results and Discussion

*Aza*-pinacol coupling of **1** with Zn in aqueous medium afforded a mixture of dibenzylamine, *R*,*S*-**2**, and *R*,*R*-**2** in a 5.25:1:1 ratio.^4^ Upon workup, the isomers were separated. One isomer is solid at room temperature, while another is liquid. The XRPD pattern of the solid isomer can be described in a monoclinic lattice with unit cell parameters as follows: *a* = 14.8195(5) Å, *b* = 5.37950(14) Å, *c* = 14.0437(3) Å, β = 95.6579(20)°, and sp.gr. **P**21/**n** (14). The crystal structure refinement was carried out with the GSAS^16,17^ program suite. Isotropic thermal displacements of all C and N atoms in the structure have been constrained to one parameter and refined. Restraints with a weight of 10.0 were used to keep carbon–carbon, carbon–nitrogen, and carbon–hydrogen atoms at distances of 1.45, 1.49, and 0.96 Å, respectively. The reliability (*wR*_p_, *R*_p_, *R*(*F*^2^)) parameters of the fit are *wR*_p_ = 3.53%, *R*_p_ = 2.24%, and *R*(*F*^2^) = 6.69% (Table 1).

**Table 1:** Atomic Coordinates and Isotropic Thermal Parameters for *R*,*S*-**2** Obtained after Structure Refinement

atom	*x*/*a*	*y*/*b*	*z*/*c*	*U*_iso_ × 100 (Å^2^)
C	0.4248(8)	0.6485(1)	0.7524(9)	3.13(5)
C	0.3474(5)	0.5103(1)	0.7123(5)	3.13(5)
C	0.3616(1)	0.3191(3)	0.6443(1)	3.13(5)
C	0.4451(4)	0.3405(8)	0.6016(4)	3.13(5)
C	0.5232(2)	0.4757(4)	0.6403(2)	3.13(5)
C	0.5162(6)	0.6159(47)	0.7264(5)	3.13(5)
C	0.3752(8)	0.5214(8)	0.0071(5)	3.13(5)
C	0.6462(1)	0.2971(5)	0.9195(8)	3.13(5)
C	0.7232(4)	0.2973(8)	0.8621(5)	3.13(5)
C	0.7949(7)	0.4666(5)	0.8937(5)	3.13(5)
C	0.7805(3)	0.6357(8)	0.9715(8)	3.13(5)
C	0.6971(2)	0.6583(7)	0.0177(7)	3.13(5)
C	0.5342(1)	0.4693(8)	0.0376(0)	3.13(5)
C	0.4111(2)	0.8251(7)	0.8340(1)	3.13(5)
N	0.4769(4)	0.7828(5)	0.9199(1)	3.13(5)

**Figure 2:**
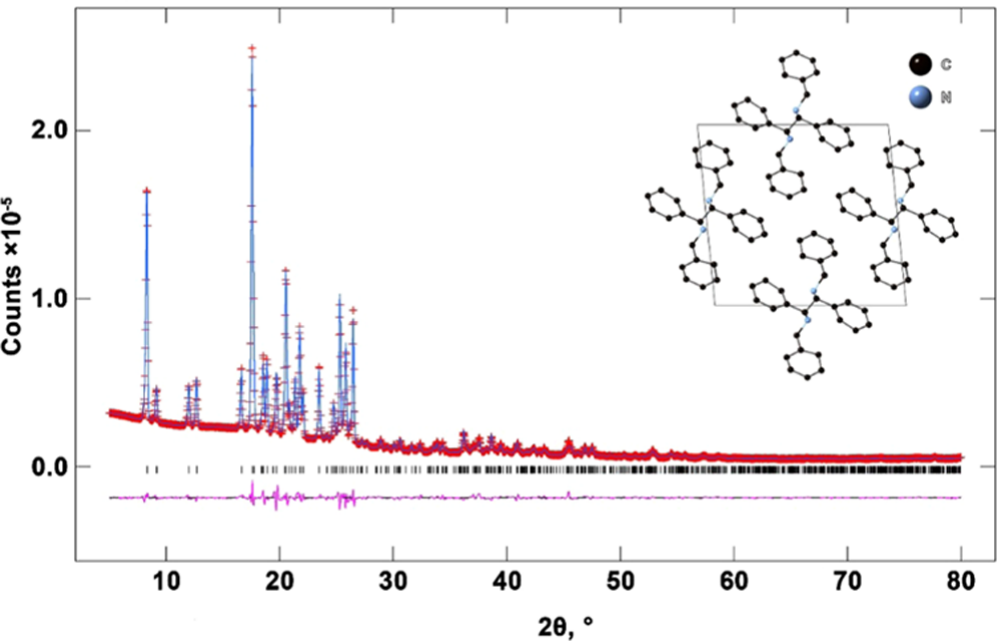
X-ray powder diffraction pattern of *R*,*S*-**2**. The red crosses are experimental points, the solid blue line is the calculated profile, and the vertical marks correspond to the positions of the Bragg reflections for the structure. The difference curve is plotted at the bottom of the figure in pink. The crystal structure of *R*,*S-***2** after the refinement of XRD data and projections onto the *ac* plane is shown in the inset. Blue and black spheres indicate N and C sites, respectively.

It is important to mention that despite the fact that the positions of the hydrogens cannot be unambiguously refined based on X-ray data, it is still possible to assign the configuration of stereocenters in the solid diastereomer. As shown in the inset of Figure 2, in the solid state, (1) two benzylic PhCH_2_ substituents and the ethanediamine linker are in a quasilinear arrangement, (2) two phenyl substituents are located on the opposite sides of the linear fragment, and (3) the tripodal fragments of the adjacent stereocenters point in opposite directions (one toward the viewer, while another opposite the viewer). Such arrangement of atoms corresponds to *R,S*-**2**.

**Figure 3:**
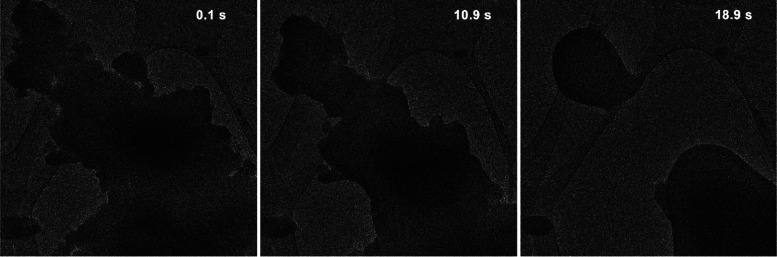
Degradation of *R*,*S***-2** within 18.9 s.

Furthermore, we attempted to investigate *R*,*S***-2** using TEM. However, the sample was not stable under the electron beam (80 kV, 5 pA), and the particles’ shape changed (probably due to melting) immediately after exposure to the electron beam, even at very low current densities (Figure 3).

**Figure 4:**
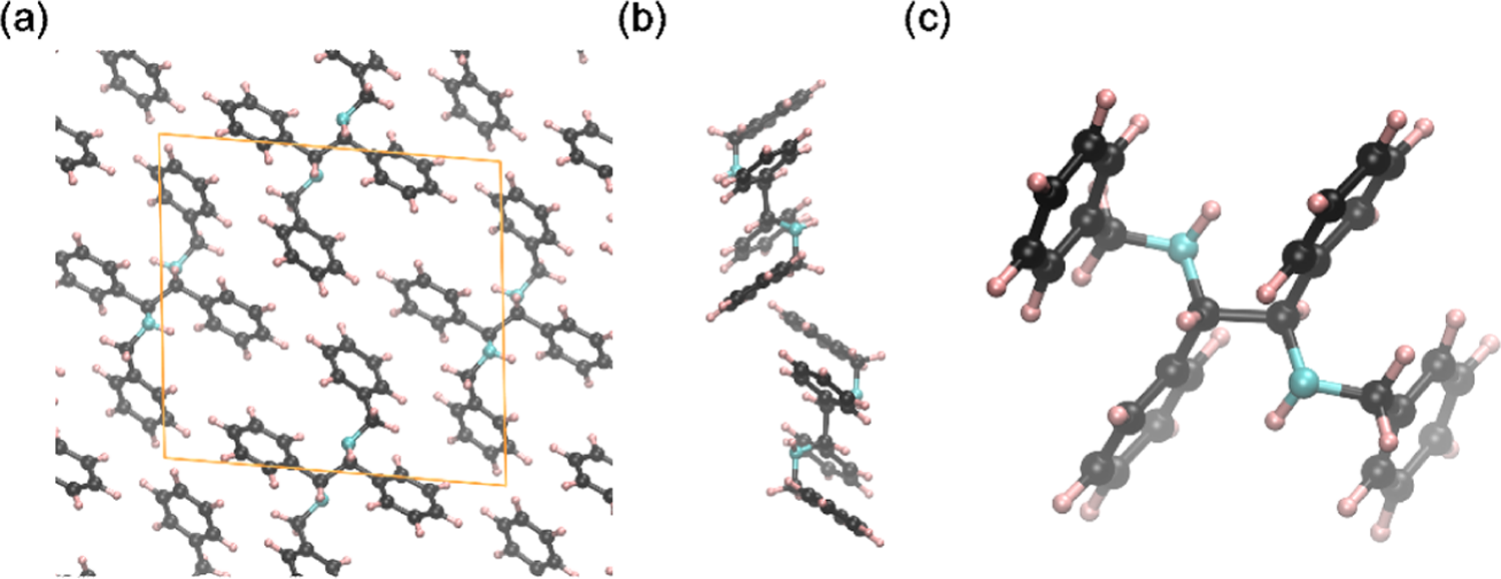
Visualization of the DFT modeling results using the same color code as in Figure 2. (a) A periodic structure. (b) Side view of the energetically optimized structure resulting from the DFT calculations. (c) Close-up of an individual molecule inside the crystal. Note that benzylic C–H protons point in opposite directions—one toward the viewer, while another opposite to the viewer. Such configuration corresponds to *R*,*S*-2.

To position the hydrogen atoms correctly, first-principle DFT calculations were performed. To obtain the final optimized structure of *R*,*S-***2** in the crystal, DFT calculations are carried out using the determined lattice parameters by XRPD. The resulting geometries are given in Figure 4, where panel (a) shows a periodic image of the optimized structure from a similar perspective as the refinement results in the inset of Figure 2. The two *R*,*S*-**2** molecules within a unit cell exhibit some tilted stacking with respect to each other, which is visualized in Figure 4b. Lastly, Figure 4c shows a close-up of the geometry and especially the relevant stereocenters of an individual molecule inside the crystal lattice. The optimization scheme has also been employed for *R*,*R*-**2** while maintaining the crystal lattice parameters. These calculations reveal that *R*,*S*-**2** is indeed thermodynamically more stable than *R*,*R*-**2** (−37.6 kJ mol^–1^) under these conditions. Therefore, the results of the XRPD calculations and DFT satisfactorily complement each other to obtain the structure and supply evidence that the synthesized crystal consists of *R*,*S*-**2** and not its *R*,*R*-analogue.

**Figure 5:**
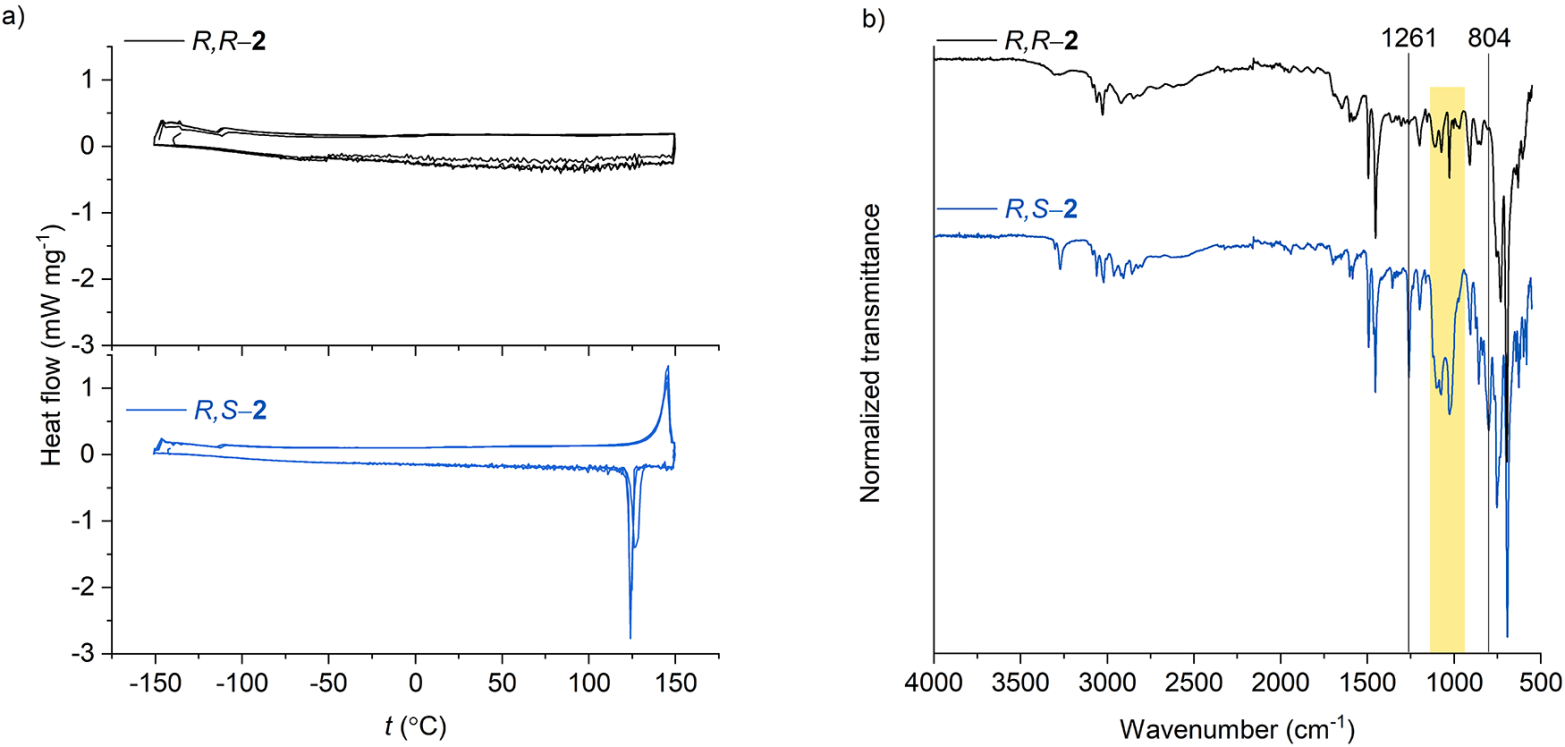
Characterization of *R*,*S*-**2** and *R*,*R*-**2**: (a) DSC traces and (b) FT-IR spectra.

DSC revealed that *R*,*S*-**2** has an m.p. of 135 °C, while *R*,*R-***2** did not crystalize upon cooling down to −150 °C (Figure 5). Several cycles of heating and cooling of *R*,*S*-**2** confirmed that the compound melts without decomposition. Both isomers possess very similar FT-IR spectra. However, vibrations at 804 and 1261 cm^–1^ are practically absent in the FT-IR spectrum of *R,R*-**2**. In addition, a stronger absorption band at 951–1131 cm^–1^ is observed for *R*,*S*-**2**. These features might serve as fingerprints of *R*,*S*-**2** in addition to different chemical shifts in ^1^H NMR spectra. Overall, our ^1^H NMR data and the fact that, at room temperature, *R*,*S*-**2** is solid and *R*,*R*-**2** is liquid agree with the data published earlier.^7,13,14^

## Conclusions

*Aza*-pinacol coupling of *N*-benzyl-1-phenylmethanimine using Zn dust affords a mixture of the *R*,*S*-diastereomer (solid, m.p. 135 °C) and the *R*,*R*-diastereomer (liquid at room temperature) in a 1:1 ratio. A combination of powder X-ray diffraction and DFT modeling revealed the configuration of stereocenters and therefore allowed us to assign the configurations of stereocenters and a specific structure to the solid diastereomer.

## Supporting Information Available

Relative atomic coordinates of optimized *R*,*S*-**2**; and ^1^H and ^13^C NMR spectra of **1**, *R*,*S-***2**, and *R*,*R*-**2** (PDF)
