# Supplementary material for: Assignment of the Crystal Structure to the Aza-Pinacol Coupling Product by X-ray Diffraction and Density Functional Theory Modeling
Source: ACS Omega. 2022 Nov 3;7(45):41581–5. doi: 10.1021/acsomega.2c05446 (PMC9670288; doi:10.1021/acsomega.2c05446)
Supplement: Supplementary file 1 — ao2c05446_si_001.pdf [file ao2c05446_si_001.pdf]

Supplementary information

Assignment of the Crystal Structure to the *Aza*-pinacol Coupling Product by X-Ray Diffraction and Density Functional Theory Modeling

Oleksandr Savateev,[1,*] Nadezda V. Tarakina,[1,*] Alexander P. Tyutyunnik[2], Salvador Martinez Rivadeneira,[3] Julian Heske[1,3], Thomas D. Kühne[3]

[1]Max-Planck Institute of Colloids and Interfaces, Department of Colloid Chemistry, Research Campus Golm, 14476 Potsdam, Germany

[2]Institute of Solid State Chemistry, Ural Branch of the Russian Academy of Sciences, 91 Pervomayskaya str., 620990, Ekaterinburg, Russia

[3]Dynamics of Condensed Matter and Center for Sustainable Systems Design, Chair of Theoretical Chemistry, University of Paderborn, Warburger Str. 100, D-33098, Paderborn, Germany.

Corresponding authors: Oleksandr.Savatieiev@mpikg.mpg.de; Nadja.Tarakina@mpikg.mpg.de

Table S1. Relative atomic coordinates of optimized *R,S*–**2** (a = 14.82 Å, b = 5.38 Å, c = 14.04 Å, α = γ = 90.0° and β = 95°)

Element	x/a	y/b	z/c
C	0.41162	0.58173	0.75314
C	0.36058	0.37294	0.72702
C	0.38296	0.21958	0.65330
C	0.45773	0.27638	0.60620
C	0.50989	0.48321	0.63246
C	0.48747	0.63500	0.70565
C	0.37155	0.49927	0.01748
C	0.65006	0.31587	0.91984
C	0.73174	0.31641	0.87981
C	0.79456	0.50252	0.90273
C	0.77547	0.68651	0.96637
C	0.69357	0.68443	0.00626
C	0.54447	0.48570	0.03359
C	0.38870	0.74792	0.83190
N	0.46005	0.74066	0.91070
H	0.30121	0.33379	0.76275
H	0.34174	0.06163	0.63152
H	0.47589	0.15780	0.54962
H	0.56810	0.52523	0.59560
H	0.52869	0.79184	0.72873

H	0.60300	0.16739	0.90133
H	0.74584	0.17061	0.83118
H	0.85805	0.50346	0.87196
H	0.82360	0.83086	0.98671
H	0.68023	0.82795	0.05618
H	0.32270	0.69896	0.85387
H	0.38415	0.93928	0.80603
C	0.08738	0.08059	0.75420
C	0.13864	0.87094	0.77853
C	0.11826	0.71650	0.85272
C	0.04455	0.77032	0.90192
C	0.99323	0.98052	0.87918
C	0.01428	0.13485	0.80613
C	0.12990	0.99197	0.49111
C	0.84958	0.82403	0.57276
C	0.76860	0.83155	0.61394
C	0.70707	0.02121	0.59109
C	0.72717	0.20420	0.52734
C	0.80921	0.19822	0.48736
C	0.95818	0.99327	0.46322
C	0.10651	0.24884	0.67402
N	0.04098	0.23942	0.58986
H	0.19641	0.83089	0.74034
H	0.15996	0.55835	0.87293
H	0.02750	0.64849	0.95798
H	0.93632	0.02279	0.91791
H	0.97357	0.29578	0.78694
H	0.89613	0.67338	0.58839
H	0.75338	0.68743	0.66283
H	0.64388	0.02595	0.62244
H	0.68007	0.35137	0.50730
H	0.82542	0.34282	0.43915
H	0.17261	0.20938	0.65118
H	0.10833	0.43988	0.69981
C	0.58968	0.41819	0.24758
C	0.64072	0.62698	0.27371
C	0.61834	0.78034	0.34743
C	0.54356	0.72354	0.39453
C	0.49141	0.51671	0.36826
C	0.51383	0.36493	0.29507
C	0.62975	0.50065	0.98324
C	0.35123	0.68406	0.08089
C	0.26955	0.68352	0.12091
C	0.20674	0.49740	0.09800
C	0.22583	0.31341	0.03435
C	0.30773	0.31550	0.99446

C	0.45683	0.51423	0.96714
C	0.61260	0.25201	0.16882
N	0.54125	0.25926	0.09002
H	0.70008	0.66613	0.23797
H	0.65955	0.93830	0.36921
H	0.52541	0.84212	0.45110
H	0.43320	0.47470	0.40513
H	0.47261	0.20808	0.27200
H	0.39829	0.83254	0.09940
H	0.25546	0.82932	0.16954
H	0.14325	0.49647	0.12876
H	0.17770	0.16907	0.01401
H	0.32107	0.17198	0.94454
H	0.67860	0.30096	0.14685
H	0.61714	0.06064	0.19469
C	0.91328	0.91926	0.24616
C	0.86201	0.12891	0.22183
C	0.88240	0.28335	0.14765
C	0.95611	0.22953	0.09844
C	0.00743	0.01933	0.12118
C	0.98638	0.86500	0.19423
C	0.87075	0.00791	0.50925
C	0.15107	0.17585	0.42760
C	0.23205	0.16832	0.38642
C	0.29359	0.97865	0.40927
C	0.27348	0.79566	0.47303
C	0.19144	0.80165	0.51300
C	0.04247	0.00661	0.53714
C	0.89414	0.75102	0.32634
N	0.95967	0.76045	0.41050
H	0.80425	0.16896	0.26002
H	0.84070	0.44150	0.12744
H	0.97316	0.35136	0.04238
H	0.06434	0.97706	0.08245
H	0.02708	0.70407	0.21342
H	0.10453	0.32651	0.41198
H	0.24728	0.31243	0.33753
H	0.35678	0.97390	0.37792
H	0.32057	0.64849	0.49306
H	0.17523	0.65705	0.56121
H	0.82805	0.79047	0.34918
H	0.89232	0.55997	0.30056
H	0.54607	0.63963	0.08464
H	0.45358	0.89097	0.95300
H	0.96133	0.15780	0.41724
H	0.97802	0.27153	0.60769

H	0.45523	0.36030	0.91609
H	0.54772	0.10895	0.04772
H	0.03932	0.84207	0.58312
H	0.02264	0.72833	0.39267

NMR spectra of compounds

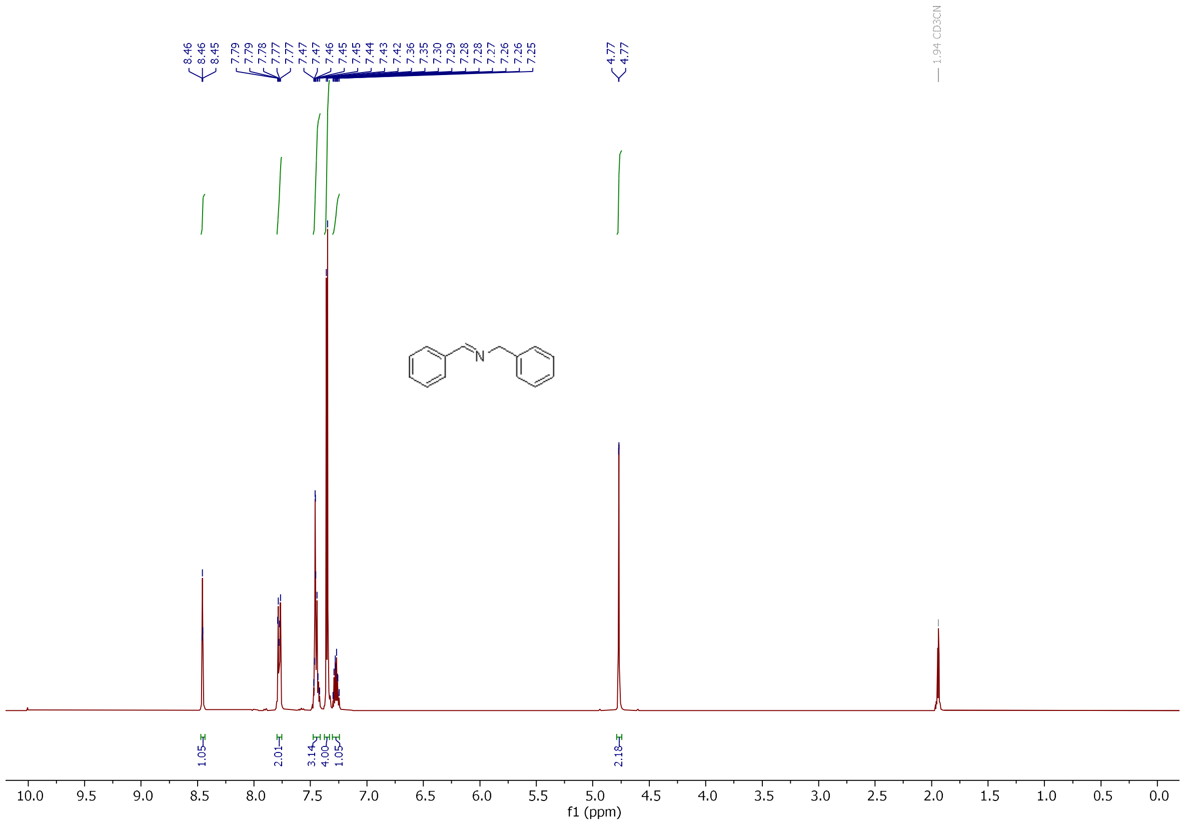

Figure S1. ^{1}H NMR spectrum of **1**.

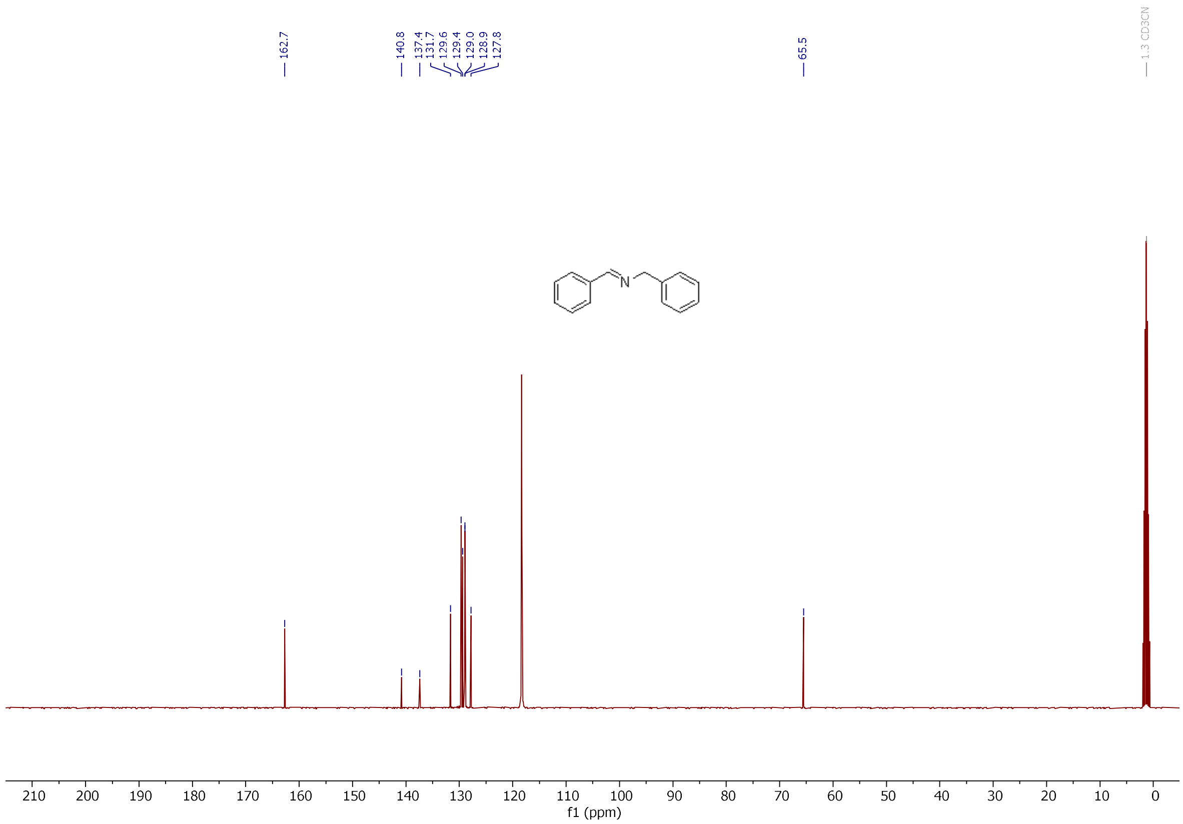

Figure S2. ^{13}C NMR spectrum of **1**.

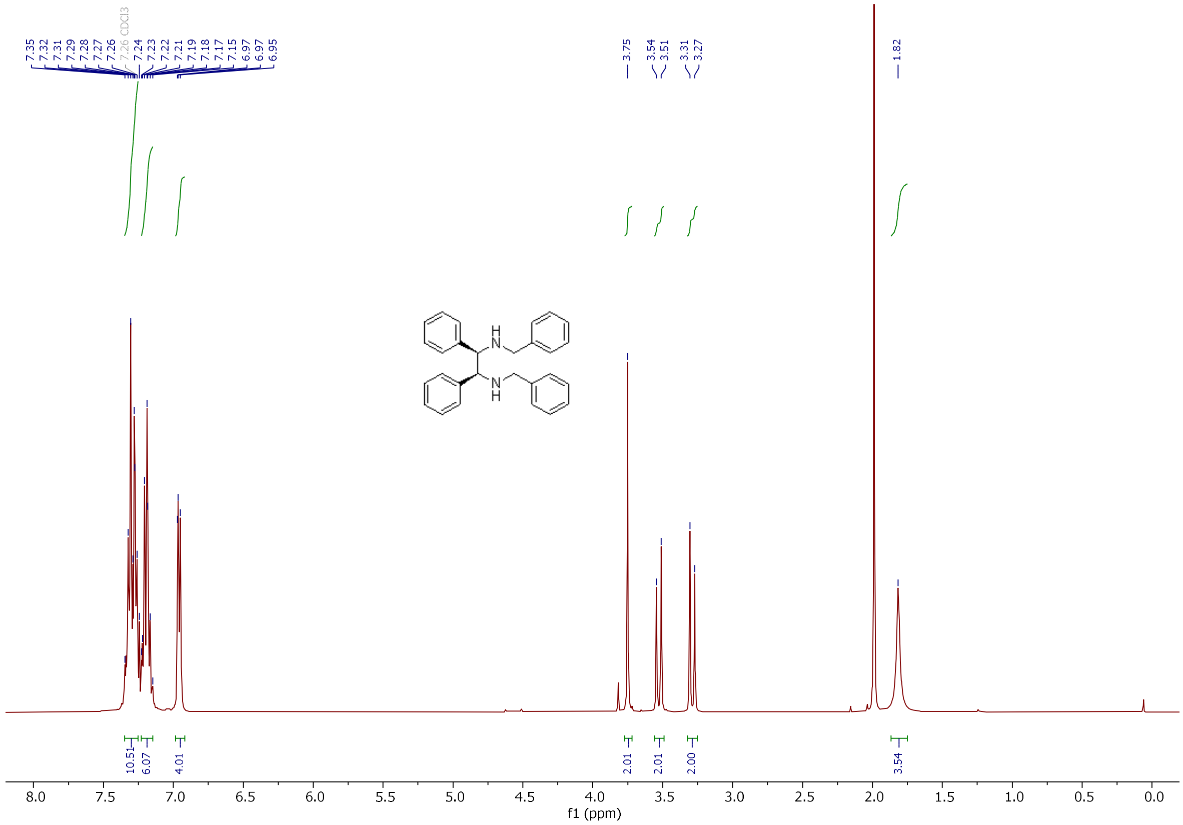

Figure S3. ^{1}H NMR spectrum of *R,S*–**2**.

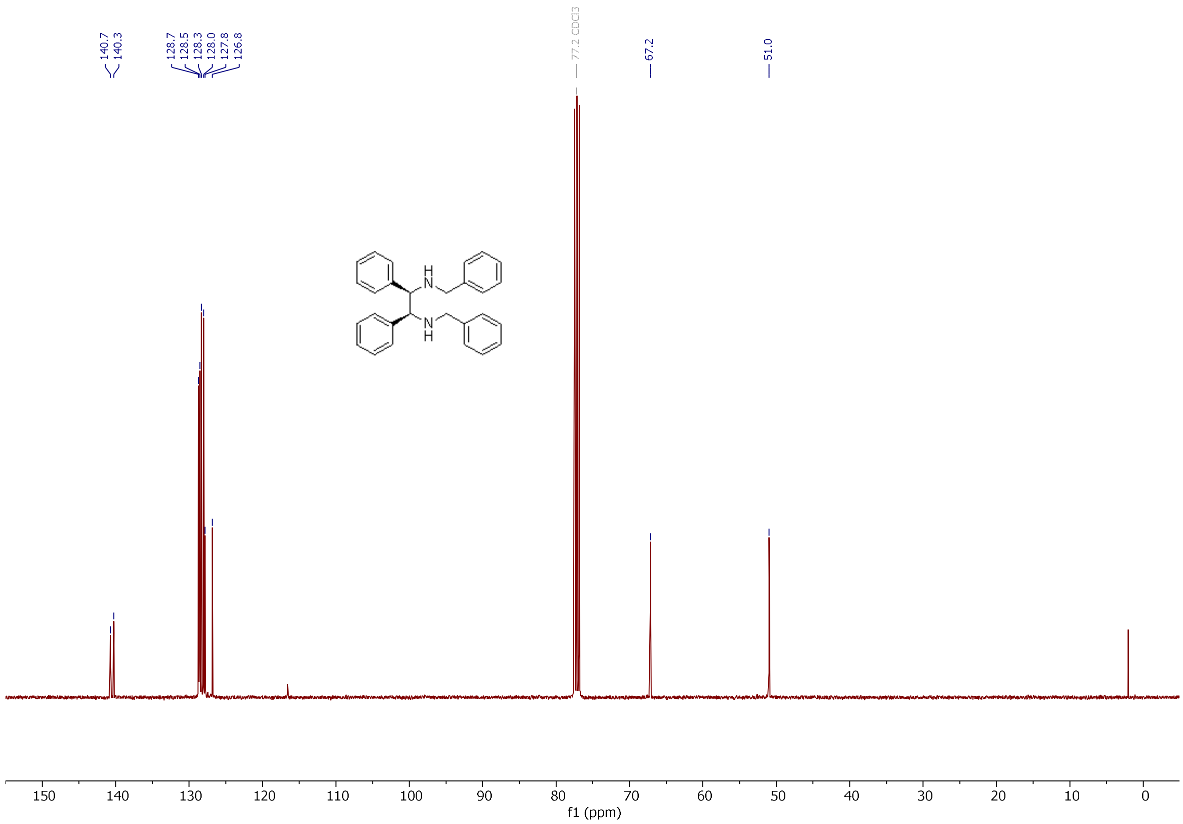

Figure S4. ^{13}C NMR spectrum of *R,S*–**2**.

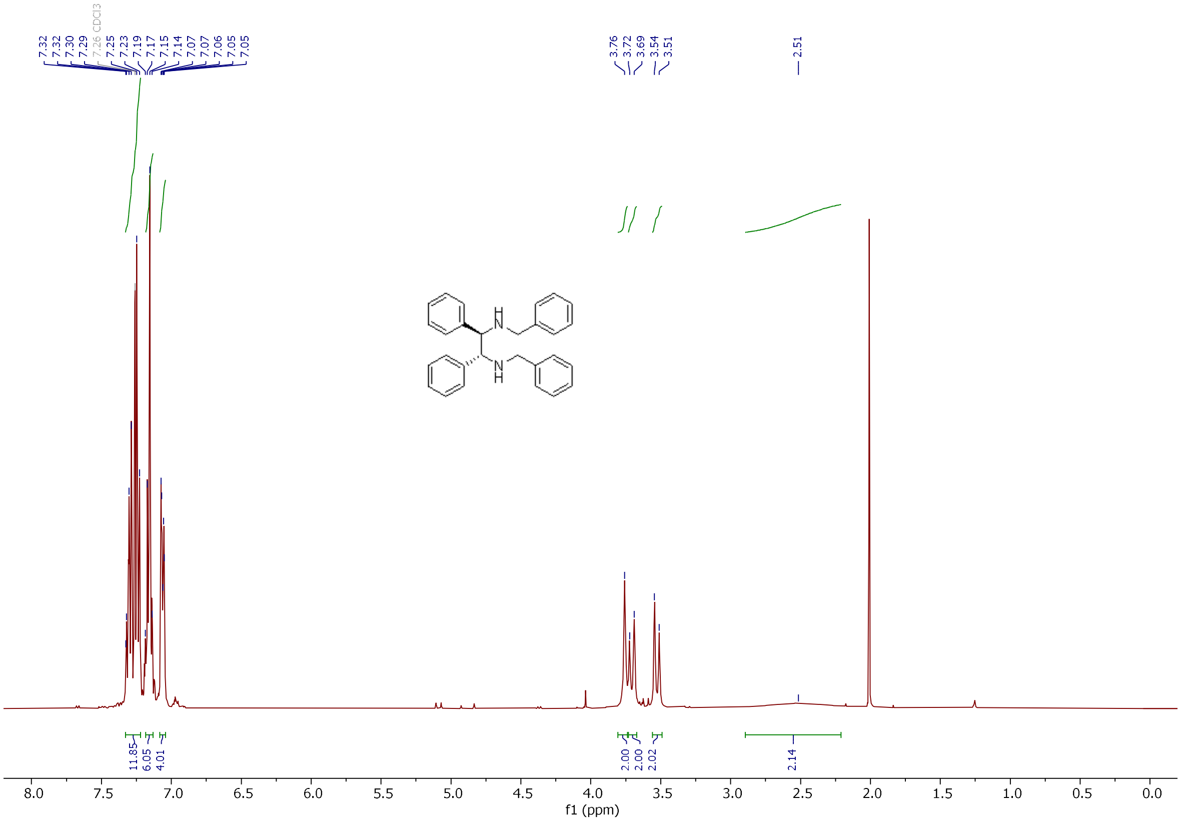

Figure S5. ^{1}H NMR spectrum of *R,R*–**2**.

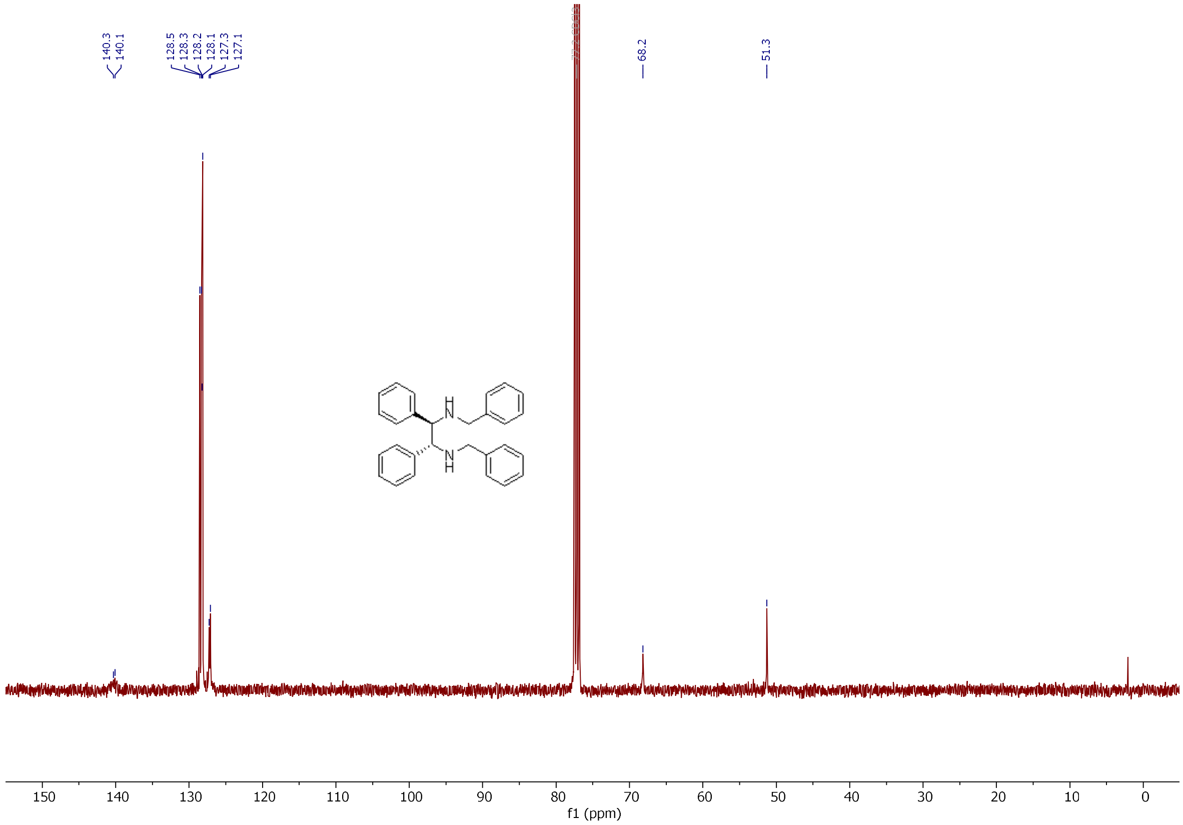

Figure S6. ^{13}C NMR spectrum of *R,R*–**2**.
